# Chinese herbal medicine for vascular cognitive impairment in cerebral small vessel disease: A protocol for systematic review and meta-analysis of randomized controlled trials

**DOI:** 10.1097/MD.0000000000022455

**Published:** 2020-10-02

**Authors:** Xinyang Zhang, Xuemei Liu, Ruyu Xia, Nannan Li, Xing Liao, Zhigang Chen

**Affiliations:** a Department of Neurology; b Central Laboratory, Dongfang Hospital; c Centre for Evidence-Based Chinese Medicine, Beijing University of Chinese Medicine; d Institute of Basic Research in Clinical Medicine, China Academy of Chinese Medical Sciences, Beijing, China.

**Keywords:** cerebral small vessel disease, chinese herbal medicine, meta-analysis, systematic review, vascular cognitive impairment

## Abstract

**Background:**

Cerebral small vessel disease (CSVD) is the most common etiology of vascular cognitive impairment (VCI). VCI in CSVD (CSVD-VCI) shows a progressive course with multiple stages and is also associated with dysfunctions such as gait, emotional and behavioral, and urinary disturbances, which seriously affect the life quality of elderly people. In mainland China, Chinese herbal medicine (CHM) is clinically used for CSVD-VCI and presenting positive efficacy, but the evidence revealed in relevant clinical trials has not been systematically evaluated. The purpose of this study is to assess the current evidence available for the clinical efficacy and safety of CHM for CSVD-VCI.

**Methods:**

A literature search of published RCTs up to April 30, 2020, has been conducted in the following 7 electronic databases: PubMed, Embase, the Cochrane Library, Chinese National Knowledge Infrastructure Database (CNKI), Chinese Science and Technology Journals Database (VIP), Wanfang Database, and Chinese Biomedical Literature Service System (SinoMed). For unpublished studies, 2 clinical trial online registration websites will be searched: ClinicalTrials.gov and Chinese Clinical Trial Registry (ChiCTR). Only randomized controlled trials (RCTs) using CHM in the treatment of patients with CSVD-VCI, which compares CHM with no treatment, placebo, or other conventional treatments, will be included in this systematic review. Primary outcomes will be set as acknowledged scales measuring cognitive function. Secondary outcomes will involve activities of daily living, behavioral, and psychological symptoms, global performance of dementia, neurological function, biological markers of endothelial dysfunction, the clinical effective rate, and adverse events. After screening studies and extracting data, the Cochrane Collaborations tool for assessing risk of bias will be applied to assess the methodological quality of included RCTs. Review Manager Version 5.3 software will be used for data synthesis and statistical analysis. Subgroup analyses, sensitivity analyses, and meta-regression will be conducted to detect potential sources of heterogeneity. The funnel plot and Eggers test will be developed to evaluate publication bias, if available. We will perform the Grading of Recommendations Assessment, Development and Evaluation (GRADE) system to appraise the quality of evidence.

**Results:**

Evidence exhibited in this systematic review will provide practical references in the field of CHM treating CSVD-VCI. Moreover, our detailed appraisals of methodological deficiencies of relevant RCTs will offer helpful advice for researchers who are designing trials of CHMs in the treatment of CSVD-VCI.

**Conclusion:**

The conclusion about the clinical efficacy and safety of CHM for CSVD-VCI will be provided for clinical plans, decisions, and policy developments in the full version of this systematic review.

**Systematic review registration:**

INPLASY202080120.

## 1 Introduction

### 1.1 Description of the condition

Cerebral small vessel disease (CSVD) refers to a series of prevalent neurological pathological processes with multiple etiologies, which affects cerebral small vessels consisting of small arteries, arterioles, capillaries, and small veins, and is highly associated with aging.[^1^,^2^] The pathological causes of CSVD are classified as arteriosclerosis (or age-related and vascular risk-factor-related small vessel diseases), cerebral amyloid angiopathy, genetic small vessel diseases distinct from cerebral amyloid angiopathy, inflammation- and immune-mediated small vessel diseases, venous collagenosis, and others, among which the first form is the most frequent.[^2^,^3^] In the field of neuroimaging, CSVD is marked by recent small subcortical infarcts, lacunes, white matter hyperintensities (WMHs), enlarged perivascular spaces, microbleeds, and brain atrophy.^[1]^ In the elderly population, the prevalence rate of WMH, lacunes, and microbleeds are separately around 50% to 98%, 8% to 28%, and 5%,[^4^,^5^,^6^] and these rates increase with age.[^7^,^8^] All these individual neuroimaging markers of CSVD are inter-related, and their respective correlations with cognition have been illustrated,[^9^,^10^,^11^,^12^] indicating the crucial role that CSVD plays in cognitive decline and dementia.

Globally accounting for 20% of all stroke cases, 45% of dementia, and over 70% of vascular dementia (VaD), CSVD is the most leading vascular cause of dementia, a major contributor to mixed dementia, and the most common etiology of vascular cognitive impairment (VCI, together with VaD as a whole are coded as 6D81 in International Classification of Diseases, 11th Revision, ICD-11).[^2^,^13^,^14^] Contrary to VCI that occurs acutely after a large vessel stroke, VCI in CSVD (CSVD-VCI) is seen as 1 with various stages and a progressive course, which presents executive dysfunctions, attention decline, set-shifting disabilities, lower efficiency of information processing in early stages, and may eventually develop into dementia with associated memory deficits; therefore, it is a category of cognitive impairment that might benefit from prevention and intervention.[^2^,^15^] Two non-mutually exclusive hypotheses about the pathogenesis of CSVD-VCI have been proposed.^[16]^ Firstly, it results from the destruction of frontal-subcortical circuits by lacunar infarcts or deep white matter lesions. Secondly, disruptions caused by deep white matter lesions occur in white matter fibers essential for cognition and emotion. Initiation and frontal executive function are mainly affected, since widespread lesions of the white matter preferentially disrupt long association fibers. In addition to cognitive decline, clinical manifestations of CSVD-VCI are gait difficulties, depression, urinary disorders, parkinsonian symptoms, pseudobulbar palsy, and limited activities of daily living, which raise the incidence of falls, disability, and death in elderly people, and devastate the lives their carers and families.[^13^,^17^,^18^,^19^,^20^]

With the aging of the population, the overall severity of CSVD-VCI and its consequent public health burden keep rapidly mounting; therefore, the development of its treatment strategy has become particularly important and attracted more and more attention.[^21^,^22^] Nowadays conventional treatments for CSVD-VCI, which include getting rid of unhealthy lifestyles, managing cerebrovascular risk factors, cholinesterase inhibitors (such as donepezil, rivastigmine, galantamine), memantine, selective calcium channel blockers with mainly vascular effects (such as nimodipine) and psychostimulants and nootropics (such as citicoline, piracetam) are aiming at slowing down the progress of disease and improving symptoms.[^23^,^24^,^25^,^26^,^27^] However, limitations of these treatments such as frequent contraindications, side effects, and high prices are unavoidable, plus there is not enough evidence to support any of them. As a result, patients resort to alternative medicine, especially Chinese herbal medicine (CHM).

### 1.2 Description of the intervention

By CHM we refer to the collection of herbal medicines used under the guidance of traditional Chinese medicine theory, an ancient holistic system of healing based on notions of harmony and balance, and adopting ideas of prevention and moderation. CHMs have been favored because they are processed from natural plants, and treating diseases with them has been considered as a relatively natural, safe, and convenient alternative medicine that saves patients money. In retrospect, CHMs have rich histories of protecting memory dysfunctions of various kinds of age-related disorders.[^28^,^29^,^30^,^31^] Several individual CHM-derived interventions with plentiful practical experience have been systematically analyzed for their competences to resist cognitive declines, such as Gingko Biloba extract and acetylcholinesterase inhibitor Huperzine A.[^32^,^33^] Currently, CHMs are widely used in modern hospitals in China, to treat cognitive impairment and enhance life quality, and their use is predicted to keep expanding in China and other countries.[^34^,^35^]

Using alone or as the adjuvant therapy to conventional treatments, CHM has received considerable investigating interest in the process of exploring better treatment strategies for various subtypes of VCI.[^36^,^37^,^38^] When compared with conventional treatments for VaD, a systematic review indicated that there were significant advantages shown in CHM groups in terms of Mini-Mental State Examination (MMSE), the Activities of Daily Living (ADL), Hasegawas dementia scale (HDS), clinical effective rate, and also fewer adverse events. Besides, when CHMs served as the adjuvant therapy to conventional treatment, patients would gain additive anti-VaD profit on MMSE scores.^[39]^ In another systematic review focusing on senile VaD, the results demonstrated that cognitive impairment was improved, plus immediate response and living quality were enhanced, when CHM was using as an adjunctive therapy.^[34]^ When participants are confined to VCI not dementia, compared with conventional treatments, CHMs still display advantages in improving MMSE and Montreal Cognitive Assessment (MoCA) scores, though the reliability of these advantages is limited by methodological insufficiencies by included trials.^[40]^ Recently, by a Cochrane Library systematic review published in 2018, 7 CHMs were respectively suggested to have potentially large benefits for VaD patients, when compared with ’no treatment’ and conventional treatments.^[41]^ However, none of these systematic reviews exclusively included CSVD-VCI participants, which means that the evidence for introducing CHMs to patients suffering CSVD-VCI is still inadequate.

### 1.3 How the intervention might work

Therapeutic mechanisms of CHMs improving VCI have been demonstrated. Firstly, CHMs inhibit the inflammatory and oxidative reactions in neurons, and thus reduce neuronal apoptosis. By markedly decreasing nicotinamide adenine dinucleotide phosphate oxidase 2 expression and raising superoxide dismutase 3 expression, Lingyang Gouteng Decoction relieves oxidative stress, and accordingly strengthens exploratory, learning and memory abilities of rats with VaD, reduces cerebral vascular or neural pathological changes.^[42]^ Isolated from Rehmanniae Radix, Rehmannioside A attenuates cognitive impairments of rats with VaD through its anti-oxidative, anti-inflammatory, and anti-apoptosis effects, which is evident by the activation of nuclear erythroid related factor-2 and the inactivation of nuclear factor-κB and Caspase-3.^[43]^ Additionally, Bushen Huoxue Decoction, Honokiol, Tanshinol have also been reported to treat VaD through anti-inflammatory and anti-oxidative mechanisms.[^44^,^45^,^46^] Secondly, CHMs enhance vascular endothelial function and promote angiogenesis. Shouwu Yizhi Decoction augments the angiogenesis by upregulating vascular endothelial growth factor (VEGF)-induced miR210 expression to stimulate Notch pathway, and further weakens neuron damage and ameliorates endothelial function, ultimately improving the cognition and memory of rats with VaD.^[47]^ As another classic CHM formulation, Taohong Siwu Decoction also promotes angiogenesis of VaD rats by inducing VEGF activities.^[48]^ Some other CHM formulations have been reported to reinforce memory capacities of VaD rats by the up-regulation of calcium signaling pathways, like Tongqiao Huoxue Decoction and Buyang Huanwu Decoction.[^49^,^50^] It should be noted that all these findings are based on animal models of VCI caused by large vessel occlusion or stenosis, since models that convincingly reflect pathological changes of human CSVD-VCI are scarce.[^51^,^52^] But they at least pictured a positive research prospect for us, as CSVD is also closely affected by inflammation and endothelial dysfunction.^[53]^

### 1.4 Why it is important to do this review

Considering the high prevalence of CSVD-VCI and the fact that conventional treatments are not ideally effective, it is necessary to identify whether CHM is beneficial for CSVD-VCI patients. In recent years, randomized controlled trials (RCTs) investigating CHMs for CSVD-VCI have been designed and implemented continuously. These RCTs have suggested diverse results of CHMs adjusting cognition, activities of daily living, and other aspects of patients, without a systematic review to pool their results and assess their methodological quality. Although RCTs, especially if double-blind, provide the most rigorous form of evidence, the top ladder in the hierarchy of evidence is represented by a combination of systematic reviews of RCTs and meta-analyses of data extracted from RCTs.^[54]^ Moreover, a growing number of medical reports illustrate hepatotoxicity and other adverse events associated with herbal drugs,[^55^,^56^] which causes social concern. So far, evidence of efficacy and safety of CHMs used to treat CSVD-CVI has not been well established. Therefore, to offer a comprehensive reference for future clinical plans, decisions, and policy developments about CSVD-VCI, this systematic review is important.

## 2 Objectives

To evaluate the efficacy and safety of CHMs used to treat CSVD-CVI.

## 3 Methods

This study protocol has been registered in the International Platform of Registered Systematic Review and Meta-analysis Protocols (INPLASY). INPLASY registration number: INPLASY202080120. This protocol is guided by the statement of preferred reporting items for systematic reviews and meta-analysis protocols (PRISMA-P).^[57]^ The full version of this systematic review will be guided by the statement of preferred reporting items for systematic reviews and meta-analyses (PRISMA).^[58]^

### 3.1 Criteria for considering studies for this review

#### 3.1.1 Types of studies

Only peer-reviewed, full-reported randomized controlled trials (RCTs) will be included in this systematic review. The reporting language of included studies will be confined to English and Chinese. Duplicate studies and studies incorrectly reporting data will be excluded.

#### 3.1.2 Types of participants

Participants of included studies should be diagnosed with CSVD-VCI. Up till now, there is still no globally recognized diagnostic criteria for CSVD-VCI, and therefore, our review will include patients diagnosed with CSVD-VCI according to diagnostic criteria in the international randomized controlled trials[^59^,^60^,^61^,^62^] or Clinical Practice Guideline for Cognitive Impairment of Cerebral Small Vessel Disease proclaimed by Geriatric Neurology Group, Chinese Society of Geriatrics in 2019.^[63]^ Briefly speaking, these diagnostic criteria contain 3 key points: subjective cognitive decline and objective evidence of cognitive impairment, neuroimaging evidence of CSVD, and identification of CSVD as evidence of cognitive impairment (namely, exclusion of cognitive impairment caused by other diseases). In particular, included studies should pay attention to the cognitive change of patients through detecting their cognitive function before and after treatments, and use at least 1 globally standardized and validated clinical scale to quantify this change, as one of the outcomes. Our review does not limit the baseline characteristics of participants enrolled by original trials. We are interested in any race, country, age, gender, education status, and admission route.

#### 3.1.3 Types of interventions

##### 3.1.3.1 Experimental interventions

Eligible experimental interventions are CHMs, and there is no limitation on types of CHMs (such as different ingredients, formulations, dosages, and routes of administration). Any CHM preparation (such as decoction, granule, oral liquid, ointment, capsule, and injection) will be taken into account. Specifically, the intervention can be CHM used alone or along with conventional treatments. Conventional treatments should be different from CHMs and used according to the clinical guideline or consensus of CSVD-VCI, such as adjustments of living habits, management of traditional risk factors of cerebrovascular disease (such as antihypertensive, hypolipidemic, and antiplatelet treatments), anti-dementia treatments like cholinesterase inhibitors, nimodipine, memantine, citicoline, DL-3-n-butylphthalide, cognitive training, routine care, etc.[^23^,^24^,^25^,^26^,^27^] On the other hand, trials from which patients of the intervention group were treated by any other traditional Chinese medicine treatments (such as acupuncture, massage, Baduanjin exercise, Taichi exercise, etc.) will be excluded.

##### 3.1.3.2 Control interventions

The eligible comparators are no treatment, placebo, or conventional treatments distinct from CHMs. Co-intervention will be allowed as long as it is used in both groups. If available, qualified comparisons will be:

1. CHM alone vs no treatment.
2. CHM alone vs a CHM placebo.
3. CHM alone vs a conventional treatment.
4. CHM combining with a conventional treatment vs the same conventional treatment alone.
5. CHM combining with a conventional treatment vs a CHM placebo combining with the same conventional treatment.
6. CHM combining with a conventional treatment placebo vs a CHM placebo combining with the conventional treatment.

#### 3.1.4 Types of outcome measures

##### 3.1.4.1 Primary outcomes

As the primary outcome of this review, we will report cognitive function, if it is measured by globally acknowledged evaluation scales such as Mini-Mental State Examination (MMSE), Montreal Cognitive Assessment (MoCA), Vascular Dementia Assessment Scale-cognitive subscale (VADAS-cog), Alzheimer's Disease Assessment Scale-cognitive subscale (ADAS-cog), Hasegawa Dementia Scale (HDS), Wechsler Memory Scale (WMS), Clock Drawing Test (CDT), the National Institute of Neurological Disorders and Stroke-Canadian Stroke Network (NINDS-CSN) Neuropsychological Assessment, or any other standardized and validated evaluation scales (as shown in Table 1).

**Table 1:**
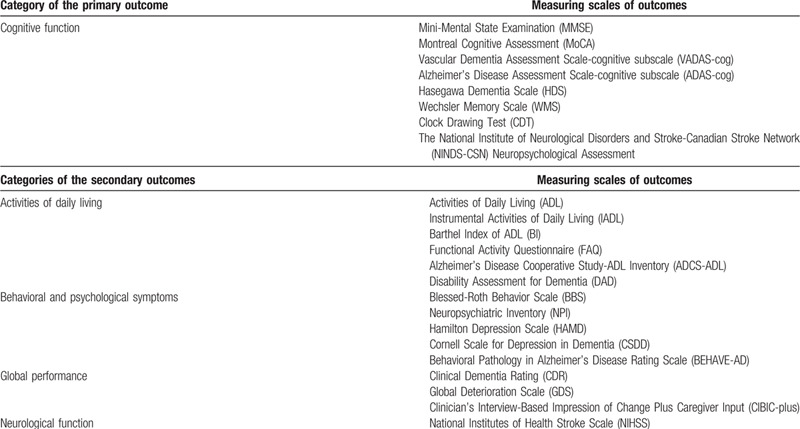
Measuring scales of primary and secondary outcomes.

##### 3.1.4.2 Secondary outcomes

Based on recognized clinical manifestations of CSVD-VCI patients, if available, secondary outcomes of this review will include the following aspects: activities of daily living, behavioral and psychological symptoms, global performance of dementia, and neurological function. These aspects will be reported, if they are measured by scales shown in Table 1, or any other standardized and validated evaluation scales. Endothelial failure, evidenced by increased levels of markers for endothelial dysfunction in the blood of patients, has been reported to lead to CSVD through various mechanisms;^[53]^ therefore, biological markers of endothelial dysfunction such as homocysteine and high-sensitive C-reactive protein, if available, will also be considered as secondary outcomes. The clinical effective rate, which means using the unified standard to estimate the effective rate of CHMs, will be displayed as another secondary outcome if possible. It is calculated by dividing the number of effective cases judged by the result of a specific outcome by the total number of cases in the same study group. Further, adverse events, including unintended disabilities, diseases, symptoms, and signs, such as abnormal liver and kidney functions and ECGs, will be reported as a secondary outcome, to demonstrate the safety of CHMs.

### 3.2 Search methods for identification of studies

#### 3.2.1 Electronic searches

We will follow the advice of a clinical librarian and a clinical expert in the field of cerebrovascular disease, in order to choose reasonable electronic bibliographic databases, and determine the search strategy and consequent concrete terms. For acquiring potentially eligible trials exploring the efficacy and safety of CHMs in the clinical treatment of CSVD-VCI, our search will be implemented in major English databases consisting of PubMed, EMBASE, and the Cochrane Library, as well as in major Chinese databases including Chinese National Knowledge Infrastructure Database (CNKI), Chinese Science and Technology Journals Database (VIP), Wanfang Database, and Chinese Biomedical Literature Service System (SinoMed), from their respective inception to April 30, 2020. No limits of the performing time or publication date of original studies will be imposed on our search. The results of searches will be continuously updated until the complete system review is published.

The search strategy that guides the selection of search terms is made up of the following 2 parts: CSVD and CHM. These terms will then be translated and applied in the search of Chinese databases. The selection of searching fields of title, abstract, or keywords will be different depending on the characteristics of databases. (Taking detailed search terms and steps used in PubMed as an example, our search strategy is shown in Table 2).

**Table 2:**
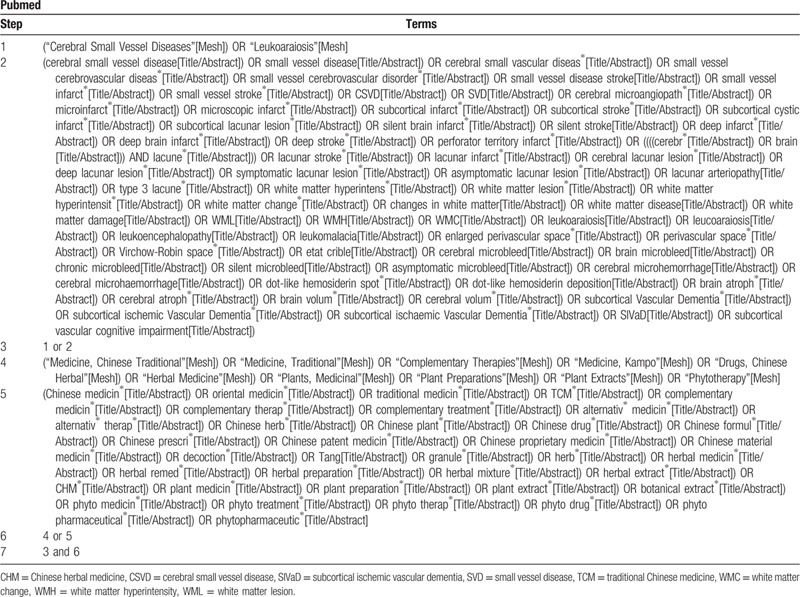
Search strategy.

#### 3.2.2 Searching other resources

To obtain additional trials that meet the inclusion criteria, we will manually examine the reference lists of relevant reviews and preliminary included trials. Besides, for ongoing or unpublished trials corresponding to our topic, we will exercise a search on clinical trial online registration websites: Clinical Trials.gov and Chinese Clinical Trial Registry (ChiCTR).

### 3.3 Data collection and analysis

#### 3.3.1 Selection of studies

**Figure 1:**
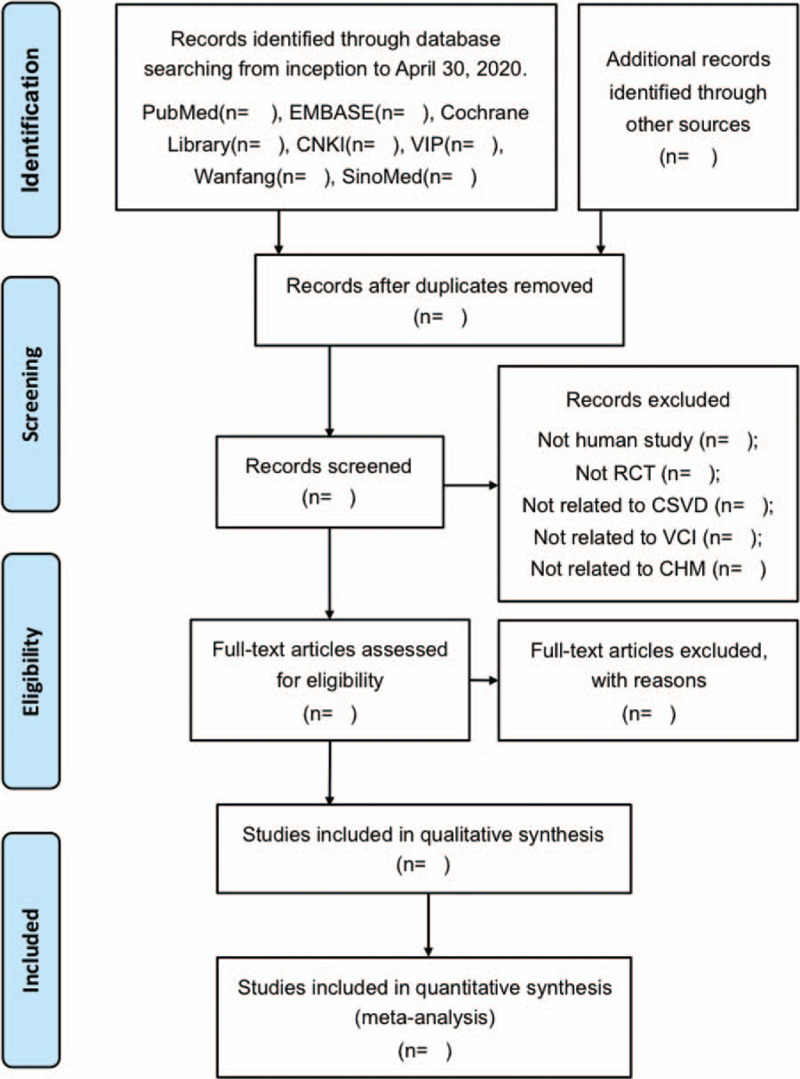
Flow diagram of study selection. CHM = Chinese herbal medicine, CNKI = Chinese National Knowledge Infrastructure Database, CSVD = cerebral small vessel disease, RCT = Randomized controlled trial, SinoMed = Chinese Biomedical Literature Service System, VCI = vascular cognitive impairment.

We will use EndNote X9 software to record and manage electronic literature from databases mentioned and complete the de-duplication process. Two review authors (XZ and XL) will independently perform the study selection process: Firstly, in order to identify studies that potentially meet the requirements of predetermined eligibility criteria, titles and abstracts that have been selected in our search process will be examined, and irrelevant ones will be excluded. Secondly, the full text of preliminary qualified studies will be retrieved and screened. If there is more than 1 report from the identical trial that meets our requirements, we will check their full text and include only 1 of them. Finally, based on the full text, the final eligibility of studies will be determined. Reasons for excluding articles at the full-text screening stage will be recorded. Any discrepancy between these 2 authors on the eligibility of a certain study will be reasonably solved by discussion and consultation with a third experienced reviewer. The study selection process is shown in a flow chart instructed by PRISMA, please see Figure 1.

#### 3.3.2 Data extraction and management

Two authors (XZ and NL) independently use standardized, pre-tested forms, which were designed by Microsoft Excel for Mac 2011 software, to extract data from included studies for managing data, assessing the quality of studies, and synthesizing needed information. Forms of data extraction focus on

1. General information of studies, including titles, the first authors name, year of publication, and performing area.
2. Methodological information, including study design, randomization, blinding, etc.
3. Characteristics of participants, including inclusion and exclusion criteria, detailed diagnose of CSVD and VCI, sample size, sex, age, disease duration, education level, risk factors of cerebrovascular disease, etc.
4. Elaborations of interventions and comparators, including the name, type and preparation, drug combination, dosage, duration of treatments, etc.
5. Outcome measures, including data of primary and secondary outcomes, measuring methods, adverse events, etc.
6. Risk of bias domains.

Any disagreement between these 2 authors will be settled through discussion and consultation with a third experienced review author. If some data needed by this systematic review is missing, we will try to contact the original research team of the included study and obtain them, through email or telephone.

#### 3.3.3 Assessment of risk of bias in included studies

The Cochrane Collaborations tool for assessing risk of bias will be independently applied by 2 review authors to assess the risk of bias of included studies, for the following domains:^[64]^ sequence generation and allocation concealment (selection bias), blinding of participants and personnel (performance bias), blinding of outcome assessors (detection bias), incomplete outcome data (attrition bias), selective outcome reporting (reporting bias), and other sources of bias (such as funding bias, conflict of interest, baseline balance). In this procedure, each domain will be classified into 3 categories (“low risk”, “high risk”, or “unclear risk”) according to the description of included studies. The overall risk of bias of an included study will be reported as “low” only if all mentioned domains are classified into “low risk”. Otherwise, the overall risk of bias for this study will be reported as “high”. Predictably, as CHM interventions are difficult to blind participants, performance bias will be common in all included studies. If these 2 authors disagree on the assessment of a certain study, it will be solved by discussion and consultation with a third experienced author.

#### 3.3.4 Measures of treatment effect

If the outcomes needed are dichotomous data, we will figure out the relative risk (RR) with 95% confidence intervals (CIs) and p values for them. Meanwhile, the weighted mean difference (WMD) and its 95% CI will be calculated when processing with continuous data collected using the consistent clinical evaluation scale. The standardized mean difference (SMD) and its 95% CIs will be calculated when processing with continuous data collected using the inconsistent clinical evaluation scale. We will pay attention to the time points at which data of outcomes were collected and reported.

#### 3.3.5 Dealing with missing data

For missing data or information, we will try to contact the original research team of included studies for supplements, mostly the first author and corresponding author, by email or telephone. After attempts to communicate, if necessary data of outcomes is still unavailable, the intention-to-treat analysis will be conducted when achievable. If the description of randomization sequence generation and allocation concealment is inadequate, which is common in RCTs published in Chinese,^[65]^ we will also try to obtain related protocols from clinical trial registration websites for the detailed description. For a methodology where descriptions are not available, a judgment of unclear risk of bias will be established.

#### 3.3.6 Assessment of heterogeneity

Heterogeneity of effect sizes among included studies will be assessed statistically using both the chi-squared test and the *I*-squared test. Data will be pooled quantitatively when at least 2 studies were available within acceptable heterogeneity, or where heterogeneity could be explained by predefined subgroup analysis. A fixed-effects model will be considered to estimate pooled effect when *I* ^2^ ≤ 50%, otherwise, a random-effects model will be used. For observed heterogeneity, we will explore potential sources of its significance through the subgroup analysis, sensitivity analysis, and meta-regression.

#### 3.3.7 Assessment of reporting biases

When at least 10 trials are included in the meta-analysis, funnel plots derived from RevMan V.5.3 software will be performed to assess publication biases. Further, Eggers tests from Stata V.12.0 software will be developed for conducting exploratory analyses, if asymmetry of the funnel plot is observed by visual inspections. Potential causes of asymmetric funnel plots will be reasonably analyzed and interpreted.

#### 3.3.8 Data synthesis

Following the recommendations of The Cochrane Collaboration, we will run Review Manager Version 5.3 software to carry out data synthesis if there are sufficient similar studies available. For dichotomous outcomes, we will obtain the RR and then aggregate the data. For continuous outcomes, if they are measured by the consistent clinical evaluation scale, we will pool WMDs between the treatment arms at the end of the follow-up, otherwise, we will pool SMDs. Where a meta-analysis is inaccessible, we will construct a table to display data.

#### 3.3.9 Subgroup analysis and investigation of heterogeneity

If available, subgroup analysis will be predefined according to following aspects: varied demographic characteristics (such as average age, course of the disease, vascular risk factors, etc.), types of CSVD based on neuroimaging standards, diagnostic criteria, severities and diagnoses of cognitive impairment of participants at baseline, comparison types, durations of the intervention and follow-up, routes of administration, and preparation forms.

#### 3.3.10 Sensitivity analysis

If possible, we will conduct sensitivity analyses to challenge the robustness of the pooled effects when there were clinically meaningful differences in primary outcomes considering: multi-center vs single-center, trials without high or unclear risk of bias either in sequence generation or allocation concealment domains vs all included trials, placebo used vs not used, reported loss-to-follow-up vs not reported, assumed worst plausible case results for patients in intervention groups with missing data.[^66^,^67^]

#### 3.3.11 Summary of findings and assessment of the certainty of the evidence

Performing the Cochrane Collaboration Network GRADE (Grading of Recommendations Assessment, Development and Evaluation), 2 review authors (XZ and RX) will separately assess the quality of evidence attributed to primary outcomes of this systematic review. Five aspects will be examined: limitations in study design, imprecision, inconsistency, indirectness, and publication bias. The quality of evidence will be categorized as high, moderate, low, or very low in this process, during which any disagreement between these 2 authors will be resolved with a third experienced author through discussion and consultation. The findings of this assessment will be portrayed as a “summary of finding” table, and it will be created in http://www.guidelinedevelopment.org/. We will make comments and explanations where necessary to help readers comprehend this review.

## 4 Discussion

Nowadays the demand for CHMs with the approved safety profile is increasingly noticeable among practitioners and public health care systems, to help patients with CSVD-VCI and thus construct a more effective and safer comprehensive treatment strategy for this disease. Therefore, findings of this systematic review will be disseminated in a peer-reviewed journal for publication, and may consequently provide references for clinical practice, consumption choices of patients, policy developments, and future research in the field of CSVD-VCI and traditional and alternative medicine. Hopefully, detailed assessments of methodological characteristics and deficiencies of included RCTs presented in the full version of this systematic review can provide helpful advice for researchers who are planning or designing clinical trials of CHMs in the treatment of CSVD-VCI.

Following this present protocol, based on RCTs, our systematic review and meta-analysis will sequentially perform appraisal processes and eventually form an elaborate summary of existing evidence focusing on the efficacy and safety of CHMs in the treatment of patients with CSVD-VCI. Primary merits making this study more persuasive include an extensive search for trials from English and Chinese databases, and also a strict evaluation of the quality of trials included. However, sources of potential heterogeneity should be noted. First of all, there are still no internationally unified diagnostic criteria for CSVD-VCI. Besides, differences in methodological quality of included studies will be prone to set up obstacles for study comparisons. Accordingly, planned subgroup analyses and sensitivity analyses will be conducted for these possible heterogeneous situations. Furthermore, foreseeable limitations of this systematic review include, firstly, unpublished studies that indicate the negative efficacy of CHMs and grey literature will be difficult to find, which may produce some biases. Secondly, secondary outcomes, such as blood indices, may not be completely reported. Thirdly, merely screening studies from English and Chinese databases will lead to the neglect of studies reported by other languages.

## Author contributions

Contributors Xinyang Zhang, Xing Liao, and Zhigang Chen conceived this study. This protocol was drafted by Xinyang Zhang, and revised by Xing Liao and Zhigang Chen. Xinyang Zhang, Xuemei Liu, Ruyu Xia, Nannan Li will separately work for study selection, quality assessment, and data extraction and synthesis.

**Conceptualization:** Xinyang Zhang, Xing Liao, Zhigang Chen.

**Data curation:** Xinyang Zhang, Xuemei Liu.

**Formal analysis:** Xinyang Zhang.

**Funding acquisition:** Xing Liao, Nannan Li

**Investigation:** Xinyang Zhang, Xuemei Liu, Nannan Li.

**Methodology:** Xing Liao, Ruyu Xia

**Software:** Xinyang Zhang, Ruyu Xia.

**Supervision:** Zhigang Chen.

**Writing – original draft:** Xinyang Zhang.

**Writing – review and editing:** Xing Liao, Zhigang Chen
